# Safety and efficacy of sorafenib in Japanese patients with hepatocellular carcinoma in clinical practice: a subgroup analysis of GIDEON

**DOI:** 10.1007/s00535-016-1204-2

**Published:** 2016-04-22

**Authors:** Masatoshi Kudo, Masafumi Ikeda, Tadatoshi Takayama, Kazushi Numata, Namiki Izumi, Junji Furuse, Takuji Okusaka, Masumi Kadoya, Satoshi Yamashita, Yuichiro Ito, Norihiro Kokudo

**Affiliations:** 1 Department of Gastroenterology and Hepatology, Kinki University School of Medicine, 377-2 Ohno-Higashi, Osaka-Sayama, Osaka 589-8511 Japan; 2 Department of Hepatobiliary and Pancreatic Oncology, National Cancer Center Hospital East, Chiba, Japan; 3 Department of Digestive Surgery, Nihon University School of Medicine, Tokyo, Japan; 4 Gastroenterological Center, Yokohama City University Medical Center, Kanagawa, Japan; 5 Department of Gastroenterology and Hepatology, Musashino Red Cross Hospital, Tokyo, Japan; 6 Department of Internal Medicine, Medical Oncology, School of Medicine, Kyorin University, Tokyo, Japan; 7 Department of Hepatobiliary and Pancreatic Oncology, National Cancer Center Hospital, Tokyo, Japan; 8 Department of Radiology, Shinshu University School of Medicine, Nagano, Japan; 9 Medical Affairs Oncology and Hematology, Bayer Yakuhin, Ltd., Osaka, Japan; 10 Hepato-Biliary-Pancreatic Surgery Division, Department of Surgery, Graduate School of Medicine, University of Tokyo, Tokyo, Japan

**Keywords:** Hepatocellular carcinoma, Sorafenib, Japanese, GIDEON

## Abstract

**Background:**

GIDEON was a prospective, global, non-interventional study evaluating the safety of sorafenib in patients with unresectable hepatocellular carcinoma in real-world practice. The aim of this subgroup analysis was to assess the safety and efficacy of sorafenib as used by Japanese patients.

**Methods:**

In Japan, 508 patients were valid for safety analysis. Efficacy and safety were evaluated by the Child-Pugh score.

**Results:**

The number of patients with Child-Pugh A and B was 432 (85.0 %) and 58 (11.4 %), respectively. The median overall survival time and time to progression in patients with Child-Pugh A and Child-Pugh B were 17.4 and 4.9 months, 3.7 and 2.3 months, respectively. The most common drug-related adverse events (AEs) included hand-foot skin reaction (47.8 %), diarrhea (35.8 %) and hypertension (24.2 %). The incidences of all or drug-related AEs were similar between patients with Child-Pugh A and B. However, all or drug-related serious AEs, AEs resulting in permanent discontinuation of sorafenib and deaths were observed more frequently in patients with Child-Pugh B compared with Child-Pugh A. Duration of treatment tended to be shorter as the Child-Pugh score worsened.

**Conclusions:**

Sorafenib was well tolerated by Japanese HCC patients in clinical settings. Patients with Child-Pugh B had shorter duration of treatment and higher incidence of SAEs. It is important to carefully evaluate patients’ conditions and assess the benefit and risk before making a decision to treat patients with sorafenib.

**Electronic supplementary material:**

The online version of this article (doi:10.1007/s00535-016-1204-2) contains supplementary material, which is available to authorized users.

## Introduction

Hepatocellular carcinoma (HCC) is the second-leading cause of cancer-related death in men and the sixth in women worldwide [1, 2]. The major risk factors for HCC are hepatitis C virus (HCV), hepatitis B virus, alcohol consumption, non-alcoholic steatohepatitis and diabetes mellitus [3, 4]. The majority of cases of HCC (70–90 % of cases) develop as a consequence of cirrhosis [5]—consequently, many patients have liver dysfunction and a high comorbidity rate. Not surprisingly, heterogeneity in the etiology, clinical symptoms and behavior of HCC makes it difficult to manage [6].

The mortality rate associated with HCC has declined by 37 %, primarily because of increased patient surveillance [7]; however, there are still many patients with unresectable HCC. Worldwide standards for the treatment of unresectable HCC have only recently been established [7–9]. Progress made in the understanding of the molecular mechanisms involved in the development and proliferation of tumors has enabled the development of effective therapeutic agents (i.e., targeted molecular therapy) for progressive HCC [10, 11].

Sorafenib is an oral multikinase inhibitor that has an inhibitory effect on tumor growth and angiogenesis [12], and it is a first-line treatment option for unresectable HCC [13]. The effect of sorafenib on prolongation of overall survival (OS) has been demonstrated in two previous phase 3, placebo-controlled, randomized studies [14, 15].

The Global Investigation of Therapeutic Decisions in Hepatocellular Carcinoma and Of Its Treatment With SorafeNib (GIDEON) was a prospective, global, non-interventional study conducted under the guidance of the European Medicines Agency [16]. The primary objective was to evaluate the safety of sorafenib in patients with unresectable HCC under real-world practice across different geographic regions as well as in a series of subgroups; 3371 patients participated from 39 countries, including Japan. Two interim analyses and a final analysis have been performed, as specified in the protocol [17, 18]. In the first and second interim analyses, 500 and 1500 patients were followed up, respectively, for ≥4 months; in the final analysis, ≥3000 patients were followed up for ≥12 months.

In Japan, GIDEON was conducted as a specific drug use-results survey under the regulation of postmarketing surveillance. Before the start of this study, all-case postmarketing surveillance was conducted separately, as required by the Japanese Ministry of Health, Labour and Welfare [19, 20]. The aim of the all-case postmarketing surveillance was to investigate unexpected drug-related adverse events (AEs), the incidence of drug-related AEs and the factors that might affect drug safety and efficacy. Patient registration was initiated after the completion of the registration for the all-case surveillance.

It is important to assess the safety and efficacy of sorafenib in daily practice and also understand the differences in the characteristics of HCC patients between Japan and other countries.

Here we report the results of the efficacy and safety analyses of sorafenib in 517 Japanese patients who participated in GIDEON.

## Methods

### Study design and objectives

The GIDEON study included patients who were eligible for systemic therapy and for whom the decision to treat with sorafenib had been made under real-world practice. Full details of the study design have been previously published [16].Efficacy analyses included OS and time to progression (TTP) by Child-Pugh score and Barcelona Clinic Liver Cancer (BCLC) status. Incidences of all or drug-related AEs and their details by Child-Pugh score were evaluated for the safety analyses. Patient demographics and baseline characteristics, incidences of drug-related AEs, BCLC stage, median OS, TTP and treatment history at baseline were obtained by region. In addition, the relationship between the number of transcatheter arterial chemoembolization (TACE) sessions before sorafenib administration and the response rate were analyzed. Child-Pugh score at the time of sorafenib administration by the number of TACE sessions was also calculated.

This study was conducted in accordance with Good Postmarketing Surveillance Practice, the principles of the Declaration of Helsinki, and all applicable laws and regulations. The protocol was reviewed and approved by the institutional review boards of all participating study sites. All patients provided written informed consent for participation before enrollment in the study (NCT00812175).

### Patients

Patients eligible for the study were outpatients diagnosed histologically, cytologically or radiographically with unresectable HCC, had a life expectancy of ≥8 weeks and were candidates for systemic therapy. The decision to provide treatment with sorafenib was made by the patient’s physicians. The exclusion criteria were based on the local product information for sorafenib [16].

### Data collection and analytical methods

All study data were collected using the case report forms as previously reported for the study [16]. AEs were graded and other safety variables were summarized descriptively in accordance with the National Cancer Institute Common Terminology Criteria for Adverse Events version 3.0 (CTCAE). The safety analysis population included patients who received ≥1 dose of sorafenib and underwent ≥1 follow-up assessment. Patients in the intent-to-treat (ITT) population had received ≥1 dose of sorafenib.

## Results

### Patient baseline characteristics

A total of 508 patients were analyzed for safety. The patients demographic and baseline characteristics by Child-Pugh score and BCLC stage at the start of therapy are shown in Table 1.

**Table 1:** Demographic and baseline characteristics by initial dose, Child-Pugh Score and BCLC stage at start of therapy

Characteristics	Total (*n* = 508)	CP classification^a^		BCLC stage^b^			
		A (*n* = 432)	B (*n* = 58)	A (*n* = 33)	B (*n* = 162)	C (*n* = 278)	D (*n* = 9)
Patients, %	100	85.0	11.4	6.5	31.9	54.7	1.8
Sex, *n* (%)							
Male	410 (80.7)	355 (82.2)	41 (70.7)	22 (66.7)	137 (84.6)	225 (80.9)	6 (66.7)
Female	98 (19.3)	77 (17.8)	17 (29.3)	11 (33.3)	25 (15.4)	53 (19.1)	3 (33.3)
Median age, years (range)	70.0 (23–90)	70.0 (23–90)	71.5 (35–86)	74.0 (31–87)	73.0 (39–90)	69.0 (23–89)	67.0 (57–78)
Age groups, *n* (%)							
<65 years	159 (31.3)	133 (30.8)	19 (32.8)	7 (21.2)	42 (25.9)	96 (34.5)	3 (33.3)
65– < 75 years	185 (36.4)	166 (38.4)	17 (29.3)	10 (30.3)	59 (36.4)	103 (37.1)	3 (33.3)
>75 years	164 (32.3)	133 (30.8)	22 (37.9)	16 (48.5)	61 (37.7)	79 (28.4)	3 (33.3)
ECOG PS at start of therapy, *n* (%)							
0	406 (79.9)	354 (81.9)	39 (67.2)	31 (93.9)	144 (88.9)	202 (72.7)	7 (77.8)
1	87 (17.1)	65 (15.0)	17 (29.3)	1 (3.0)	16 (9.9)	66 (23.7)	1 (11.1)
2	5 (1.0)	5 (1.2)	0	0	0	4 (1.4)	0
3	1 (0.2)	0	1 (1.7)	0	0	0	1 (11.1)
TNM stage at entry of study, *n* (%)							
Stage I	14 (2.8)	12 (2.8)	0	13 (39.4)	1 (0.6)	0	0
Stage II	135 (26.6)	121 (28.0)	11 (19.0)	19 (57.6)	103 (63.6)	9 (3.2)	0
Stage IIIA	97 (19.1)	80 (18.5)	14 (24.1)	1 (3.0)	49 (30.2)	46 (16.5)	0
Stage IIIB	10 (2.0)	9 (2.1)	1 (1.7)	0	4 (2.5)	6 (2.2)	0
Stage IIIC	22 (4.3)	21 (4.9)	1 (1.7)	0	1 (0.6)	20 (7.2)	0
Stage IV	225 (44.3)	184 (42.6)	31 (53.4)	0	4 (2.5)	197 (70.9)	9 (100.0)

*BCLC* Barcelona Clinic Liver Cancer, *CP* Child-Pugh, *ECOG PS* Eastern Cooperative Oncology Group Performance Status, *TNM* tumor-node-metastasis

^a^For CP classification, 18 patients were not evaluable

^b^For BCLC stage, 26 patients were not evaluable

Median age was 70 years, and approximately 80 % of the patients were males; 85 % of the patients were Child-Pugh A and 11.4 % were Child-Pugh B; 54.7 % of the patients were classified as BCLC stage C. A worse Eastern Cooperative Oncology Group (ECOG) score correlated with a worse Child-Pugh score. The ECOG score was similar among patients with BCLC stages A and B but tended to be higher in patients with BCLC stage C.

### Sorafenib administration

Sorafenib administration by Child-Pugh score and BCLC stage at the start of therapy is shown in Table 2. Among the patients with Child-Pugh A, a similar proportion received an initial daily dose of 400 mg (47.0 %) versus 800 mg (46.3 %). A slightly higher proportion of patients with Child-Pugh B (53.4 %) than Child-Pugh A (47.0 %) received an initial daily dose of 400 mg.

**Table 2:** Study drug administration summary by Child-Pugh and BCLC stage at start of therapy

	Total (*n* = 508)	CP classification					BCLC stage			
		A, < 7 (*n* = 432)	B, 7–9 (*n* = 58)	B, 7 (*n* = 42)	B, 8 (*n* = 12)	B, 9 (*n* = 4)	A (*n* = 33)	B (*n* = 162)	C (*n* = 278)	D (*n* = 9)
Initial sorafenib dose, *n* (%)										
200 mg	21 (4.1)	20 (4.6)	1 (1.7)	1 (2.4)	0	0	3 (9.1)	10 (6.2)	7 (2.5)	0
400 mg	246 (48.4)	203 (47.0)	31 (53.4)	24 (57.1)	4 (33.3)	3 (75.0)	23 (69.7)	81 (50.0)	119 (42.8)	4 (44.4)
600 mg	8 (1.6)	8 (1.9)	0	0	0	0	0	4 (2.5)	3 (1.1)	1 (11.1)
800 mg	231 (45.5)	200 (46.3)	26 (44.8)	17 (40.5)	8 (66.7)	1 (25.0)	6 (18.2)	66 (40.7)	149 (53.6)	4 (44.1)
Average daily dose^a^, mg	419.0	425.0	400.0	400.0	584.5	400.0	400.0	400	471.0	412.0
Median treatment duration^b^, week	15.90	17.40	7.60	8.80	5.70	10.35	23.60	17.70	13.20	16.10

*CP* Child-Pugh, *BCLC* Barcelona Clinic Liver Cancer stage

^a^Determined by actual days on the study drug, excluding interruptions

^b^From initial visit to last dosing date

Of the patients with BCLC stage B, 50.0 and 40.7 % received an initial daily dose of 400 and 800 mg, respectively. The proportion of patients with BCLC stage C (53.6 %) who received an initial daily dose of 800 mg was slightly higher than for those with BCLC stage B (40.7 %).

The average daily dose of sorafenib, 419.0 mg, was similar to that received by patients with Child-Pugh A and B scores and with BCLC stages A and B (400.0 mg); that of BCLC stage C was slightly higer (471.0 mg).

The median treatment duration with sorafenib was 15.90 weeks. Treatment duration tended to become shorter as the Child-Pugh score worsened; patients with Child-Pugh A and Child-Pugh B had median treatment durations of 17.40 and 7.60 weeks, respectively.

### Efficacy analyses

**Fig. 1:**
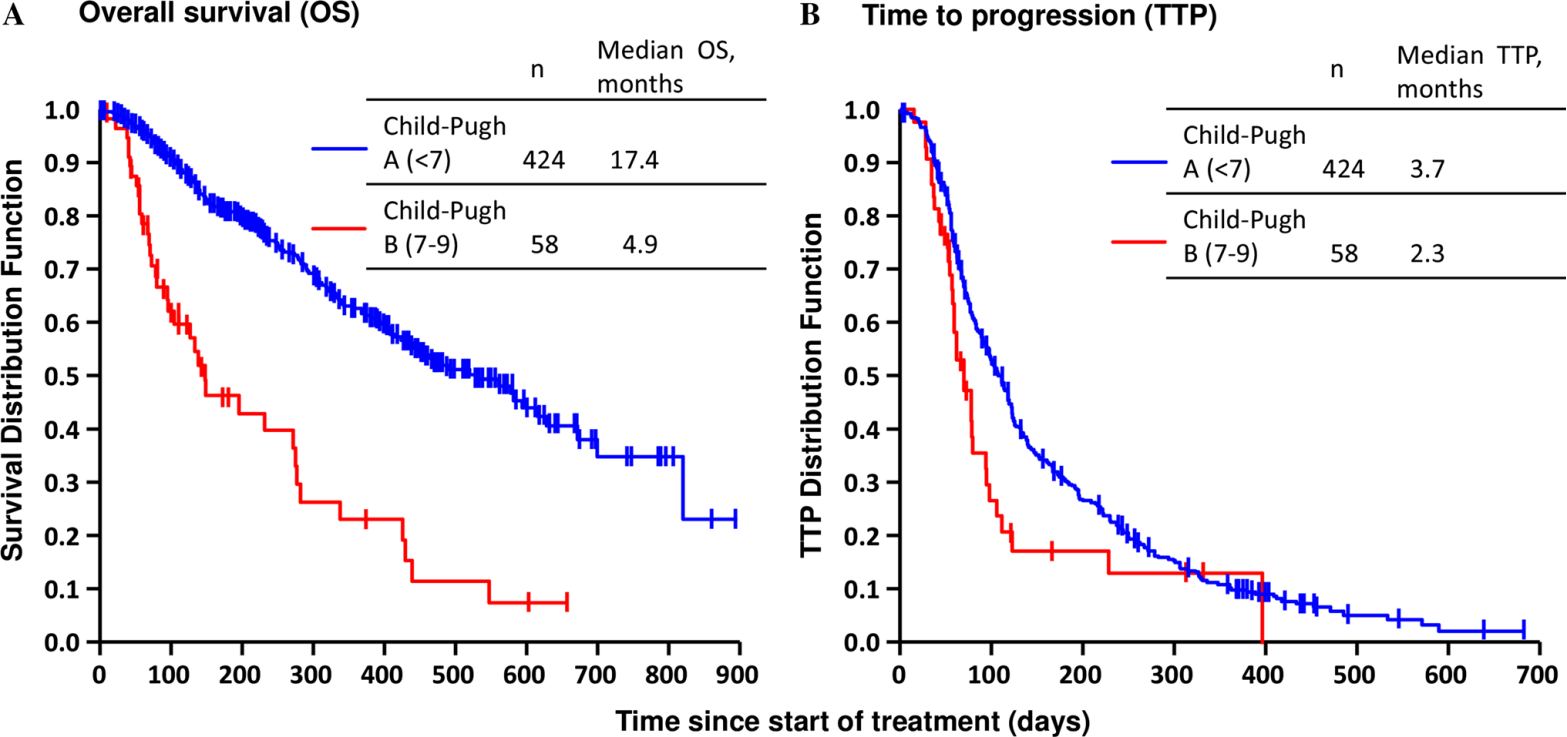
Kaplan-Meier curve of OS and TTP (intent-to-treat population): **a** OS by baseline Child-Pugh status; **b** TTP by baseline Child-Pugh status. *OS* overall survival, *TTP* time to progression

**Fig. 2:**
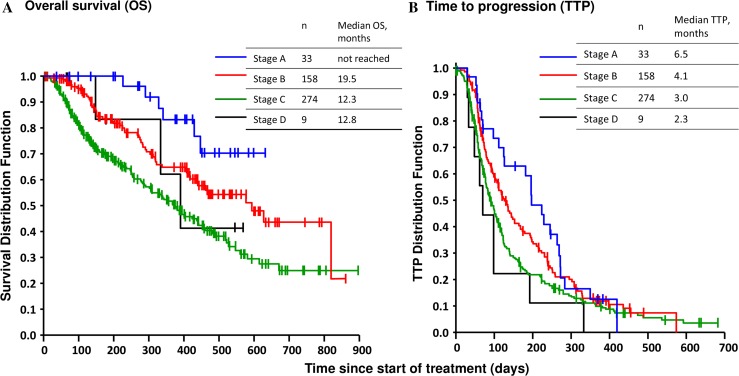
Kaplan-Meier curve of OS and TTP (intent-to-treat population): **a** OS by BCLC classifications; **b** TTP by BCLC classifications. *BCLC* Barcelona Clinic Liver Cancer, *OS* overall survival, *TTP*  time to progression

A total of 500 patients were analyzed for efficacy in the ITT analysis. The difference between the safety and ITT population was due to reasons such as exclusion of patients who had a history of sorafenib treatment. OS and TTP by Child-Pugh score and BCLC stage per Response Evaluation Criteria in Solid Tumors (RECIST) v1.0 are shown in Figs. 1 and 2.

The median OS in patients with Child-Pugh A (17.4 months; Fig. 1a) was longer than in those with Child-Pugh B (4.9 months), suggesting that the Child-Pugh score is a prognostic factor. Similarly, the median TTP in patients with Child-Pugh A (3.7 months; Fig. 1b) was longer than in patients with Child-Pugh B (2.3 months), but the difference was not as remarkable as that seen for OS. The TTP by modified RECIST (mRECIST) also showed a similar trend (data not shown).

Although the median OS in patients with BCLC stage A was not reached, OS tended to be shorter with more advanced BCLC stage (Fig. 2a). Median OS was longer in patients with better liver function; median OS in patients with BCLC stage B of Child-Pugh A and Child-Pugh B were 20.7 (95 % CI 15.4–unknown) and 8.9 (95 % CI 4.6–14.4) months, respectively. TTP, as measured by RECIST, tended to be shorter with more advanced BCLC stage (Fig. 2b); TTP in patients with BCLC stage A was 6.5 (95 % CI 4.1–8.8) months, 4.1 (3.4–5.0) months in patients with stage B and 3.0 (2.6–3.5) months in patients with stage C. TTP by mRECIST showed a similar tendency (data not shown).

### Safety analyses

A summary of AEs by Child-Pugh score and BCLC stage at the start of sorafenib therapy is shown in Table 3.

**Table 3:** Overview of safety data by Child-Pugh classification

Adverse events^a^, *n* (%)	Total (*n* = 508)	CP classification			
		A, < 7 (*n* = 432)	B, 7–9 (*n* = 58)	B, 7 (*n* = 42)	B, 8 (*n* = 12)
AEs, all grades	482 (94.9)	410 (94.9)	55 (94.8)	39 (92.9)	12 (100.0)
Drug-related AEs, all grades	445 (87.6)	381 (88.2)	50 (86.2)	36 (85.7)	12 (100.0)
AEs, grade 3 or 4	223 (43.9)	195 (45.1)	23 (39.7)	19 (45.2)	3 (25.0)
Drug-related AEs, grade 3 or 4	190 (37.4)	161 (37.3)	24 (41.4)	19 (45.2)	5 (41.7)
SAEs^b^, all grades	209 (41.1)	160 (37.0)	40 (69.0)	28 (66.7)	9 (75.0)
Drug-related SAEs^b^, all grades	90 (17.7)	70 (16.2)	19 (32.8)	16 (38.1)	3 (25.0)
AEs resulting in permanent discontinuation of sorafenib^c^	210 (41.3)	167 (38.7)	30 (51.7)	24 (57.1)	6 (50.0)
Deaths^d^	77 (15.2)	51 (11.8)	20 (34.5)	12 (28.6)	6 (50.0)
Any drug-related AEs ≥5 %, %	87.6	88.2	86.2	85.7	100.0
Hand-foot skin reaction	47.8	49.5	37.9	35.7	58.3
Diarrhea	35.8	37.3	24.1	26.2	16.7
Hypertension	24.2	25.5	15.5	16.7	16.7
Alopecia	19.5	21.3	5.2	4.8	8.3
Anorexia	19.7	18.3	20.7	21.4	25.0
Fatigue	17.7	17.6	20.7	26.2	8.3
Rash/desquamation	14.6	15.7	8.6	9.5	8.3
Hoarseness	10.8	11.3	6.9	9.5	0
Decreased platelet count	9.1	10.0	5.2	7.1	0
Pyrexia	5.7	5.6	5.2	4.8	0
Pruritus	4.7	5.1	1.7	2.4	0
Amylase increased	5.5	5.6	6.9	9.5	0
Hypophosphatemia	4.1	4.2	5.2	4.8	8.3
Vomiting	2.4	1.9	5.2	2.4	16.7
Abnormal laboratory tests	2.2	1.4	6.9	9.5	0
Incidence of hepatic system drug-related AEs (≥5 %), %					
ALT increased	7.3	7.2	6.9	2.4	25.0
AST increased	7.9	8.1	5.2	2.4	16.7
Hyperbilirubinemia	5.5	5.3	6.9	2.4	25.0
Liver dysfunction	4.5	3.7	12.1	14.3	8.3
Hypoalbuminemia	3.1	2.8	6.9	7.1	8.3
Hepatic encephalopathy	2.4	2.1	5.2	4.8	8.3
Incidence of drug-related SAE^b^ (≥2 %), %					
Any drug-related SAE	17.7	16.2	32.8	38.1	25.0
Liver dysfunction	2.2	1.4	8.6	9.5	8.3
Hepatic encephalopathy	1.4	1.2	3.4	4.8	0
Gastric ulcer	0.8	0.5	3.4	4.8	0
Abnormal laboratory tests	0.8	0.5	3.4	4.8	0

*AE* adverse event, *ALT* alanine aminotransferase, *AST* aspartate aminotransferase, *CP* Child-Pugh, *SAE* serious adverse event

^a^Graded as per the National Cancer Institute Common Terminology Criteria for Adverse Events, version 3.0

^b^An SAE/drug-related SAE is defined as any AE/drug-related AE occurring at any dose that results in any of the following outcomes: death, life-threatening illness, hospitalization or prolongation of existing hospitalization, persistent or significant disability/incapacity, congenital anomaly/birth defect or medically important event

^c^Any AEs

^d^Treatment-emergent deaths occurring up to 30 days after last sorafenib dose

The incidence of AEs and drug-related AEs in patients with Child-Pugh A and Child-Pugh B were similar (94.9 % and 94.8, 88.2 and 86.2 %, respectively)

The incidence of serious AEs (SAEs) and drug-related SAEs in patients with Child-Pugh B was higher than in patients with Child-Pugh A (69.0 % and 37.0, 32.8 and 16.2 %, respectively). The incidence of AEs leading to permanent discontinuation of sorafenib was 38.7 % in patients with Child-Pugh A and 51.7 % in patients with Child-Pugh B. The incidence of treatment-emergent death occurring up to 30 days after discontinuation of sorafenib was 11.8 % in patients with Child-Pugh A and 34.5 % in patients with Child-Pugh B.

Drug-related AEs reported more frequently in patients with Child-Pugh A than with B included hand-foot skin reaction (HFSR), hypertension, alopecia, hoarseness, decreased platelet count, pruritus and rash/desquamation. However, vomiting and abnormal laboratory tests were reported more often in patients with Child-Pugh B than with A.

The drug-related AEs of the hepatic system of liver dysfunction, hypoalbuminemia, and hepatic encephalopathy were observed more frequently in patients with Child-Pugh B than Child-Pugh A. Among drug-related SAEs, the incidence rates of liver dysfunction, hepatic encephalopathy, gastric ulcer and abnormal laboratory tests were also higher in patients with Child-Pugh B.

### Comparison with other geographic regions in GIDEON

Patient baseline characteristics, incidence of drug-related AEs, BCLC stage, median OS and TTP, and treatment history in the five geographic regions of the GIDEON study (Asia-Pacific, European Union, Latin America, USA and Japan) are summarized in Table 4 [21]. In Japanese patients, the median age was higher (70 years) and a history of locoregional therapy was also higher (84.4 %) than for other regions. Particularly, TACE was conducted more frequently in Japanese patients (71.3 %). Infection with HCV was etiologically associated with 53.1 % of HCC cases in Japan, which was comparable to the USA. Japan experienced the highest incidence of drug-related AEs, including CTCAE grades 3 and 4, drug-related SAEs and AEs resulting in permanent discontinuation of sorafenib, but the lowest rate of deaths. In Japan, 43.7 % of patients had BCLC stage A at the time of initial diagnosis, but the majority of patients had progressed to stage B (31.9 %) or C (54.7 %) by the initiation of sorafenib therapy. Regardless of BCLC stage, Japanese patients showed a longer time from initial diagnosis to death than those in other regions. In addition, the median OS from the start of sorafenib therapy was longest, but the median TTP was shorter than in other regions.

**Table 4:** Background difference by region

	Total (*n* = 3202)	Asia-Pacific (*n* = 928)	Europe (*n* = 1113)	Latin America (*n* = 90)	USA (*n* = 563)	Japan (*n* = 508)
Patients, %	100	28.9	34.8	2.8	17.6	15.9
Median (range) age, years	62 (15–98)	54 (19–87)	66 (15–94)	67 (18–98)	61 (20–87)	70 (23–90)
Daily dose, mg						
Median	688.0	800.0	780.0	800.0	527.0	419.0
Mean	616.5	663.4	668.1	748.5	555.7	487.2
Etiology, %						
Hepatitis B	36.5	82.3	18.1	3.3	14.0	24.2
Hepatitis C	32.9	5.0	35.6	35.6	54.9	53.1
Alcohol use	26.0	16.2	34.3	15.6	39.3	13.2
NASH	2.8	0.2	3.2	6.7	6.0	2.4
Treatment-emergent AEs, %						
Drug-related AEs, all grades	66.0	48.7	68.8	48.9	71.9	87.6
Drug-related AEs, grade 3 or 4	23.6	12.2	27.4	12.2	23.8	37.4
Drug-related SAEs^a^, all grades	9.3	3.4	10.9	13.3	7.5	17.7
AEs leading to permanent discontinuation of sorafenib^b^	31.4	20.2	35.1	13.3	36.2	41.3
Deaths^c^	23.7	19.1	25.7	33.3	33.4	15.2
BCLC stage at the initial diagnosis						
A	21.6	9.1	24.6	23.3	16.9	43.7
B	19.7	15.8	25.9	31.1	11.5	20.3
C	30.1	37.6	31.9	23.3	26.5	17.7
D	2.8	2.6	2.0	7.8	5.9	0.8
BCLC stage at the start of sorafenib therapy						
A	7.1	2.8	8.5	17.8	9.9	6.5
B	19.8	10.2	24.3	40.0	12.4	31.9
C	52.0	61.1	52.9	28.9	36.2	54.7
D	5.4	5.0	4.0	8.9	11.7	1.8
Median (range) time from the initial diagnosis to death, months						
BCLC stage A	59.2 (51.9–67.5)	54.0 (10.3–NA)	49.3 (42.3–58.0)	23.3 (17.2–NA)	24.9 (18.4–53.5)	91.0 (76.6–113.1)
BCLC stage B	29.9 (25.6–39.0)	31.0 (18.4–47.7)	27.3 (23.0–33.1)	22.2 (12.9–NA)	19.7 (11.1–36.8)	47.9 (40.9–86.2)
BCLC stage C	10.6 (9.4–12.4)	10.3 (8.6–13.4)	11.0 (8.9–13.0)	11.2 (3.1–NA)	8.5 (6.2–10.2)	27.7 (16.6–40.8)
BCLC stage D	8.9 (6.2–13.1)	8.9 (8.6–14.8)	11.0 (4.2–21.7)	NA	7.5 (4.5–12.8)	13.1 (NA–NA)
Overall	25.5 (23.9–28.3)	20.9 (17.3–25.2)	25.0 (22.9–28.7)	19.5 (13.5–NA)	14.8 (13.1–17.0)	79.6 (62.1–96.0)
Median OS from the start of sorafenib therapy, months	10.9	9.7	11.8	13.7	8.5	14.5
Median TTP from the start of sorafenib therapy, months	4.8	3.8	6.4	15.2	5.5	3.4
Median time from initial diagnosis to the start of sorafenib therapy, months	3.9	2.6	3.7	1.2	2.8	24.1
Previous therapy, %						
Surgical treatment	21.1	24.2	15.5	5.6	9.4	43.3
Transplant	2.6	3.3	2.0	2.2	4.8	0.2
All locoregional therapy	57.5	67.2	43.5	27.8	49.4	84.4
TACE	47.2	60.3	33.1	13.3	37.1	71.3
RFA	17.5	12.8	14.9	17.8	11.5	38.4
HAI	5.6	5.2	1.0	2.2	3.9	18.9
PEI	4.7	2.7	5.3	0	1.1	11.6
Systemic therapy	5.2	5.0	3.8	0	3.4	11.6

*AE* adverse event, *BCLC* Barcelona Clinic Liver Cancer, *HAI* hepatic arterial infusion chemotherapy, *NA* not applicable, *NASH* nonalcoholic steatohepatitis, *OS* overall survival, *PEI* percutaneous ethanol injection, *RFA* radiofrequency ablation, *SAE* serious adverse event, *TACE* transcatheter arterial chemo-embolization, *TTP* time to progression

^a^A drug-related SAE is defined as any drug-related AE occurring at any dose that results in any of the following outcomes: death; life-threatening condition; hospitalization or prolongation of existing hospitalization; persistent or significant disability/incapacity; congenital anomaly/birth defect; medically important event

^b^Any AEs

^c^Treatment-emergent deaths occurring up to 30 days after the last sorafenib dose

### Effects of the number of transcatheter arterial chemoembolization sessions on the tumor response rate and Child-Pugh status

The relationships between the number of TACE sessions and its tumor response rate before the start of sorafenib therapy and between the number of TACE sessions and Child-Pugh score at initiation of sorafenib therapy are shown in Table 5. It has been shown that there is no significant correlation between the tumor reduction rate (World Health Organization and RECIST criteria) and the pathologic necrosis rate after TACE with lipiodol [22]. The response evaluation criteria that take account of the tumor necrosis are thus required in liver cancer treatment. Therefore, it is common in Japan to determine the treatment effect using the modified RECIST and the response evaluation criteria in cancer of the liver [23, 24].

**Table 5:** Summary of response to TACE

TACE session	Patients, *n*	Tumor response^a^, %						CP classification, %		
		CR	Non-CR	Responder	Nonresponder	Disease control	Progressors	A	B	Not evaluable
1	362	18.5	67.1	68.2	17.4	78.4	7.2	85.4	11.0	3.6
2	286	12.2	75.2	68.5	18.9	77.6	9.8	83.2	12.9	3.8
3	219	12.8	74.9	67.1	20.6	76.7	11.0	82.6	12.3	5.0
4	161	8.1	77.0	61.5	23.6	75.8	9.3	80.7	13.0	6.2
5	112	8.9	78.6	62.5	25.0	75.0	12.5	83.9	10.7	5.4
6	75	5.3	78.7	56.0	28.0	69.3	14.7	82.7	16.0	1.3
7	47	2.1	80.8	57.4	25.5	72.3	10.6	76.6	21.3	2.1
8	33	3.0	75.8	39.4	39.4	60.6	18.2	72.7	27.3	0.0
9	21	4.8	71.4	42.9	33.3	61.9	14.3	71.4	28.6	0.0

*CP* Child-Pugh, *CR* complete response, *TACE* transcatheter arterial chemoembolization

^a^For tumor response, a total rate of each category did not reach 100 % because of the missing or unevaluable patients

The number of TACE sessions was higher in Japan than in other regions; however, patients with ≥6 TACE sessions tended to have lower complete and partial response rates. In addition, when the number of TACE sessions before sorafenib therapy was ≥6, the percentage of patients with Child-Pugh B was higher at initiation of sorafenib therapy.

## Discussion

GIDEON was a large-scale, prospective, noninterventional study with ≥3300 patients from 39 countries evaluating the safety and efficacy of sorafenib and the factors that affect decision making with regard to treatment options. The median treatment duration of sorafenib in patients with Child-Pugh B was shorter than in patients with Child-Pugh A. Although the incidence of all or drug-related AEs was similar between Child-Pugh A and B, the incidence of all or drug-related SAEs, the number of AEs resulting in permanent discontinuation of sorafenib and deaths was higher in patients with Child-Pugh B. The incidence of drug-related AEs of hepatic-related events, such as liver dysfunction, hypoalbuminemia and hepatic encephalopathy, was higher in patients with Child-Pugh B than with Child-Pugh A. The incidence of drug-related AEs was analyzed by patient-year in consideration of the treatment duration of sorafenib. The results showed that drug-related AEs and liver function in patients with Child-Pugh A and Child-Pugh B were 1.59 and 2.67, 0.07 and 0.37 events per patient-year, respectively (data not shown). It is necessary to fully weigh the benefits versus risks associated with sorafenib treatment in patients with Child-Pugh B. Furthermore, when Cox regression analysis was given for parameters used in the Child-Pugh classification (excluding hepatic encephalopathy) at the time of treatment initiation with sorafenib, albumin and bilirubin levels were identified as contributing factors to the OS, with the hazard ratio for bilirubin being the highest (data not shown). The results of global analysis had shown that ascites, albumin and bilirubin levels were factors affecting the OS, which were similar to those of the Japanese subgroup analysis [25].

Before the start of this study in Japan, all-case surveillance was conducted separately under the regulations of postmarketing surveillance [19, 20]. The incidence of drug-related AEs was 90.2 %. Frequently observed drug-related AEs included HSFR (51.4 %), liver dysfunction (26.4 %), diarrhea (25.1 %) and hypertension (21.6 %) [19, 20]. The results of Japanese subgroup analyses showed that drug-related AEs were observed in 87.6 % of patients. Frequently observed drug-related AEs included hand-foot skin reaction (47.8 %), liver dysfunction (4.5 %), diarrhea (35.8 %) and hypertension (24.2 %).

Reasons for the low incidence of liver dysfunction may be that a safety bulletin (liver failure and hepatic encephalopathy) was issued from the Japanese Proper Use Advisory Committee immediately after initiation of registration and that there was routine monitoring (e.g., periodic liver function tests followed by appropriate dose reduction or interruption) during sorafenib treatment.

The incidence of drug-related AEs of liver dysfunction was less than 1 % in the sorafenib arm in the Phase III SHARP and Asia-Pacific trial. In the SHARP trial, times to deterioration of liver function (Child-Pugh classification) were similar between the sorafenib and placebo arm (data not shown).

Compared with other regions, the mean time from the initial diagnosis to death in Japan tended to be longer irrespective of BCLC stage. This difference could be the result of early detection or because patients in Japan had more treatment opportunities than those in other regions. In addition, TTP from the start of sorafenib therapy in Japanese patients was the shortest among patients worldwide; Japan’s early monitoring by imaging appears to be the major reason why Japanese patients have the shortest TTP [26, 27].

The present results also showed that the incidence of AEs resulting to permanent discontinuation of sorafenib in Japanese patients was 41.3 %, a higher rate than seen in other regions. The incidence of HFSR was 4.1 %, which was the second highest rate after liver dysfunction (4.3 %) (data not shown). Although the HFSR itself is not a life-threatening AE, it can decrease patient quality of life, cause infection and pain, limit daily activities and lead to a complex medical situation.

It has been reported that the incidence of HFSR differs between Japanese and non-Japanese patients [28]. The incidence of hand-foot skin reaction in the all-case surveillance and in this study was high: 51.4 and 47.8 %, respectively, higher than for other Asian countries (31.7 % in Korean patients [29]). Although the discontinuation rate due to HFSR is low, the cause is not fully understood, and future studies will be needed.

Incidence of drug-related AEs was highest in Japan, but treatment-emergent death occurring up to 30 days after discontinuation of sorafenib was lowest compared with other regions. Ealier discontinuation of sorafenib treatment may be related to the apparent lower rate of treatment-emergent death.

The number of TACE sessions performed before sorafenib therapy was higher for Japanese patients than for those in other regions. Patients with ≥6 TACE sessions tended to have lower response rates, and there was a higher proportion of Child-Pugh B patients at initiation of sorafenib therapy. TACE failure/refractoriness was defined by the Japan Society of Hepatology in 2010 and revised in 2014 [30, 31], which was after patient registration began in the GIDEON study. In TACE-refractory patients with intermediate-stage HCC, the deterioration of liver function is accelerated when TACE is continued, and conversion to sorafenib significantly improves the median OS [32, 33]. Therefore, in the case of uncontrolled tumors by TACE, TACE should not be repeated and alternative treatments, such as sorafenib, are recommended.

GIDEON did not include a control group or randomization. The number of patients with Child-Pugh B was much smaller than with Child-Pugh A; therefore, the results should be interpreted with caution. Japanese patients were not registered in the Phase III SHARP and the Asia-Pacific trial [14, 15]. Thus, obtaining background information and treatment trends from real-world practice data in Japanese patients may provide a valuable contribution to the future of HCC treatment. In this subgroup analysis of Japanese patients, there was an earlier diagnosis, more frequent treatment with TACE before sorafenib therapy and a tendency toward longer OS irrespective of BCLC stage at the time of initial diagnosis compared with other regions.

In conclusion, sorafenib was well tolerated by Japanese HCC patients in clinical settings. Patients with Child-Pugh B had a shorter duration of treatment and higher incidence of SAEs. Therefore, it is critical to evaluate the patient's benefit and risk before making a decision to treat with sorafenib for patients with Child-Pugh B.

## Electronic supplementary material

Below is the link to the electronic supplementary material.

Supplementary material 1 (DOCX 24 kb)
