# Supplementary material for: Safety and efficacy of sorafenib in Japanese patients with hepatocellular carcinoma in clinical practice: a subgroup analysis of GIDEON
Source: J Gastroenterol. 2016 Apr 22;51(12):1150–60. doi: 10.1007/s00535-016-1204-2 (PMC5121182; doi:10.1007/s00535-016-1204-2)
Supplement: Supplementary file 1 — Supplementary material 1 (DOCX 24 kb) [file 535_2016_1204_MOESM1_ESM.docx]

**Supplementary Table 1. Study Sites**

| **Study site** | **Principal investigator** |
| --- | --- |
| Sapporo-Kosei General Hospital | Dr. Yoshiyasu Karino |
| Teine Keijinkai Hospital | Dr. Kunihiko Tsuji |
| Tohoku University Hospital | Dr. Takao Iwasaki |
| Yamagata University Hospital | Dr. Hisayoshi Watanabe |
| Tokyo Medical University Ibaraki Medical Center | Dr. Yasushi Matsuzaki |
| Saitama Medical University Hospital | Dr. Satoshi Mochida |
| Dokkyo Medical University | Dr. Keiichi Kubota |
| National Cancer Center Hospital East | Dr. Masafumi Ikeda |
| Chiba University Hospital | Dr. Fumihiko Kanai |
| Kyorin University Hospital | Dr. Junji Furuse |
| National Cancer Center Hospital | Dr. Takuji Okusaka |
| Medical Hospital, Tokyo Medical and Dental University | Dr. Shigeki Arii |
| Tokyo Medical University Hospital | Dr. Fuminori Moriyasu |
| Tokyo Women’s Medical University Hospital | Dr. Masakazu Yamamoto |
| The University of Tokyo Hospital, Graduate School of Medicine, Hepatobiliary-Pancreatic Surgery Division, Department of Surgery | Dr. Kiyoshi Hasegawa |
| The University of Tokyo Hospital, Department of Gastroenterology | Dr. Syuichiro Shiina |
| Japanese Red Cross Medical Center | Dr. Takuya Hashimoto |
| Nihon University Itabashi Hospital | Dr. Tadatoshi Takayama |
| Japanese Red Cross Musashino Hospital | Dr. Namiki Izumi |
| Showa University Hospital | Dr. Toshiyuki Baba |
| Yokohama City University Medical Center | Dr. Kazushi Numata |
| University of Yamanashi Hospital | Dr. Masanori Matsuda |
| Ogaki Municipal Hospital | Dr. Takashi Kumada |
| Juntendo University Shizuoka Hospital | Dr. Takafumi Ichida |
| Kanazawa University Hospital | Dr. Tatsuya Yamashita |
| Kyoto University Hospital | Dr. Takamichi Ishii |
| Kinki University Hospital, Faculty of Medicine | Dr. Masatoshi Kudo |
| Hyogo College of Medicine | Dr. Shuhei Nishiguchi |
| Ehime University Hospital | Dr. Masashi Hirooka |
| Tokushima University Hospital | Dr. Mitsuo Shimada |
| Kochi Medical School Hospital | Dr. Shinji Iwasaki |
| Social Insurance Shimonoseki Welfare Hospital | Dr. Satoyoshi Yamashita |
| Kyushu University Hospital | Dr. Yoshihiko Maehara |
| Kurume University Hospital | Dr. Takuji Torimura |
| Fukuoka University Hospital | Dr. Syoutarou Sakisaka |
| National Hospital Organization Kyushu Medical Center | Dr. Hideki Saitsu |
| Hospital of the University of Occupational and Environmental Health, Japan | Dr. Masaru Harada |
| Nagasaki University Hospital | Dr. Kazuhiko Nakao |
| Kagoshima City Hospital | Dr. Takeshi Hori |
| Kagoshima University Medical And Dental Hospital | Dr. Akihiro Moriuchi |
